# Periodontal disease, chronic kidney disease and mortality: results from the third national health and nutrition examination survey

**DOI:** 10.1186/s12882-015-0101-x

**Published:** 2015-07-07

**Authors:** Ana C. Ricardo, Ambarish Athavale, Jinsong Chen, Hemanth Hampole, Daniel Garside, Phillip Marucha, James P. Lash

**Affiliations:** Division of Nephrology, Department of Medicine, University of Illinois at Chicago, 820 South Wood Street, 418W CSN, MC 793, 60612 Chicago, IL USA; Department of Medicine, John H. Stroger Jr. Hospital of Cook County, Chicago, IL USA; Institute of Minority Health Research, University of Illinois at Chicago, Chicago, IL USA; School of Dentistry, Oregon Health and Science University, Portland, OR USA

**Keywords:** Mortality, Clinical epidemiology, Chronic kidney disease

## Abstract

**Background:**

Periodontal disease is associated with increased mortality in the general population, however its prognostic significance in chronic kidney disease (CKD) is not known. We evaluated the joint effect of periodontal disease and CKD on all-cause and cardiovascular mortality.

**Methods:**

Prospective observational study of 10,755 adult participants in the National Health and Nutrition Examination Survey, 1988–1994 (NHANES III). CKD was defined as estimated glomerular filtration rate < 60 ml/minute/1.73 m^2^ or albumin-to-creatinine ratio ≥ 30 mg/g. Periodontal disease was defined as moderate (> 4 mm attachment loss in ≥ 2 mesial sites or 5 mm pocket depth in ≥ 2 mesial sites), or severe (> 6 mm attachment loss in ≥ 2 mesial sites and > 5 mm pocket depth in ≥ 1 mesial site). All-cause and cardiovascular mortality were evaluated using Cox proportional hazards models.

**Results:**

There were 1,813 deaths over a median follow-up of 14 years. In multivariate analyses, as compared to participants with neither periodontal disease nor CKD, those with periodontal disease only or CKD only had increased all-cause mortality (HR 1.39; 95 % CI, 1.06 - 1.81 and 1.55; 1.30 - 1.84, respectively). The presence of both periodontal disease and CKD was associated with HR (95 % CI) 2.07 (1.65 - 2.59) for all-cause mortality, and 2.11 (1.52 - 2.94) for cardiovascular mortality. We found no evidence of multiplicativity or additivity between periodontal disease and CKD. In stratified analyses limited to individuals with CKD, periodontal disease (vs. not) was associated with adjusted HR (95 % CI) 1.35 (1.04 - 1.76) for all-cause, and 1.36 (0.95 - 1.95) for cardiovascular mortality.

**Conclusions:**

These findings confirm the well-established association between periodontal disease and increased mortality in the general population, and provide new evidence of this association among individuals with CKD.

## Background

Chronic kidney disease (CKD) stages 1–5 affects approximately 26 million individuals in the United States (U.S.) and the size of this population is expected to grow [1]. It is well established that individuals with CKD are at increased risk for cardiovascular morbidity and mortality compared to the general population [2, 3]. However, traditional risk factors do not fully account for the heightened cardiovascular risk in the CKD population [4]. Therefore, investigation of novel and potentially modifiable risk factors is warranted.

Periodontal disease is a common, chronic inflammatory condition of the tissues surrounding the teeth caused by accumulation of bacteria which can lead to tooth loss [5]. In the general population, periodontal disease has been associated with all-cause mortality and cardiovascular mortality [6–11],which is thought to be mediated by endothelial dysfunction caused by cytokines and inflammatory factors released in response to chronic bacterial infection in the oral cavity, ultimately leading to atherosclerosis [12]. Less is known about the association of periodontal disease with CKD. Several cross-sectional studies have reported a potential link between periodontal disease and CKD [13–15], with a potential bidirectional relationship mediated by hypertension and diabetes [16]. Similar to cardiovascular disease (CVD), chronic inflammation is considered a risk factor for CKD; therefore, it has been postulated that the increased risk for CKD in individuals with periodontal disease might be related to inflammatory cytokines (e.g., interleukin 6, tumor necrosis factor, thromboxane B_2_) which may lead to atherosclerotic changes and thrombus formation in the renal vasculature [13, 17].

We conducted a study to assess the joint effect of periodontal disease and CKD on mortality among National Health and Nutrition Examination Survey, 1988–1994 (NHANES III) participants. We hypothesized that the presence of both periodontal disease and CKD would be associated with the highest risk for all-cause and cardiovascular mortality.

## Methods

### Study population

NHANES III was a cross-sectional, stratified, clustered, multistage probability sample survey of the civilian, noninstitutionalized U.S. population, conducted by the National Center for Health Statistics (NCHS) between 1988 and 1994 [18, 19]. The survey protocol was approved by the NCHS institutional review board. All participants provided informed consent. Participants underwent a home interview followed by an extensive physical examination (including an assessment of periodontal disease as described below) and blood and urine sampling at a mobile examination center (MEC). Self-reported information on sociodemographic characteristics and presence of medical conditions was collected during the home interview. Blood pressure (BP), blood and urine samples were obtained during the physical examination.

### Exposure ascertainment

#### Periodontal measures

Periodontal measures were done on randomly assigned half-mouths, one upper quadrant and one lower quadrant selected at the beginning of the examination [19]. The buccal and mesial-buccal aspects of each tooth were scored separately for each periodontal measure: gingival bleeding, calculus, gingival recession, and pocket depth. Loss of attachment was derived from two measurements made at each site: (1) the distance from the free gingival margin to the cemento-enamel junction, and (2) the distance from the free gingival margin to the bottom of the sulcus (pocket depth). When the gingival margin had receded and the cemento-enamel junction was exposed, the first number was scored as a negative value and was an indication of gingival recession. The loss (level) of attachment variables was calculated by subtracting the recorded distance of the free gingival margin to cemento-enamel junction (1) from the recorded distance of the free gingival margin to the base of the sulcus (2). Periodontal disease was defined based on the Centers for Disease Control and Prevention (CDC) criteria [20, 21] as follows: moderate periodontal disease as at least 2 mesial sites with > 4 mm attachment loss or at least 2 mesial sites with 5 mm pocket depth (not on the same tooth), and severe periodontal disease as at least 2 mesial sites with > 6 mm attachment loss and one or more mesial sites with > 5 mm pocket depth. For the purposes of this study, individuals with either moderate or severe disease were classified as having periodontal disease.

#### Kidney function measures

CKD was defined by either an estimated glomerular filtration rate (eGFR) < 60 ml/min/1.73 m^2^, using the CKD epidemiology collaboration (CKD-EPI) equation for creatinine [22] or the presence of urine albumin-to-creatinine ratio (ACR) ≥ 30 mg/g. We used the formula for correction of serum creatinine recommended in the NHANES III Data File Documentation [23].

### Outcome ascertainment

Vital status was determined using the NHANES III Linked Mortality Public-use File, which provides vital status follow-up data in person-months from the date of the NHANES III survey participation through the date of death or 12/31/2006. Mortality was ascertained by the NCHS through a probabilistic match between NHANES III participants and National Death Index death certificate records. Participants who were not matched with any death records were considered to be alive through the follow-up period [24]. For this study, cardiovascular mortality was defined as death due to diseases of the heart, essential hypertension and hypertensive kidney disease, cerebrovascular disease, atherosclerosis, and other diseases/disorders of the circulatory system (codes I00-I99).

### Covariates definition

Race/ethnicity was categorized as non-Hispanic White, non-Hispanic Black, Mexican American, or other. Income was classified as annual family income < 20,000 or ≥ 20,000 U.S. dollars. Educational attainment was categorized as less than high school or ≥ high school. Participants were considered to have health insurance if they self-reported coverage by Medicare, Medicaid, CHAMPUS/Veterans Affairs, or any other health insurance plan over the preceding month.

Dental care use was defined as “recommended” if the last dental visit was < 1 year prior and for a routine reason, or “less than recommended” if the last dental visit was > 1 year ago, for a problem, or for another reason [15]. Participants were classified as current, past or never smoker based on responses to the questions “Have you smoked at least 100 cigarettes during your entire life?” and “Do you smoke cigarettes now?” Participants had three BP measurements at the MEC in the sitting position, after 5 minutes of rest, using a standardized protocol. [18] The averages of all systolic BP available readings are reported here. Hypertension was defined as BP > 140/> 90 mmHg or the use of antihypertensive medications. Diabetes was defined as a history of diabetes, use of insulin or other medication to treat diabetes, a fasting blood glucose ≥126 mg/dL, or a random blood glucose ≥ 200 mg/dL. Participants were considered to have CVD if they self-reported history of myocardial infarction, stroke, or congestive heart failure. Family history of CVD was defined as having a first degree relative with history of myocardial infarction at age < 50 yrs. Body mass index (BMI) was calculated as weight in kilograms divided by height in meters squared. Hemoglobin A1c (HbA1c) was measured using the Diamat Analyzer System. Serum C-reactive protein was quantified using latex-enhanced nephelometry. White blood cell count determination was made using a Coulter Counter. Fibrinogen was measured in citrated plasma using an automated coagulation analyzer [25].

### Statistical methods

NCHS recommendations were followed to account for stratification and clustering of the survey design, as well as oversampling of ethnic minorities and elderly persons [23]. Continuous variables were expressed as means (standard error) or medians (interquartile range) if not normally distributed; and categorical variables as weighted percentage. Chi-squared and Student’s t tests were used to compare categorical and continuous variables, respectively. Cox proportional hazards models were used to determine the joint effect of periodontal disease and CKD on mortality adjusting for important covariates (age, gender, race/ethnicity, education, annual income, diabetes, hypertension, smoking, CVD, BMI, family history of premature CVD, HbA1c, total cholesterol and systolic BP) which were chosen based on prior literature [10, 13, 14]. We did not include C-reactive protein, fibrinogen or white blood cell count in the regression models because markers of inflammation are thought to be in the causal pathway of the association between periodontal disease and mortality [10, 17]. We conducted formal tests for multiplicativity and additivity between periodontal disease and CKD on all-cause and cardiovascular mortality. Additional multivariable analyses were conducted to evaluate the joint effect of periodontal disease and albuminuria (ACR ≥ 30 mg/g), and of periodontal disease and low eGFR (< 60 mL/min/1.73 m^2^) on outcomes; these models included the variables listed above in addition to eGFR and natural log of urine ACR, respectively. Stratified analyses by CKD status were also conducted. All tests were two-sided, and p < 0.05 was considered significant for hypothesis testing. The proportional hazards assumption of the Cox models was examined using Schoenfeld residuals, which showed no significant departure from proportionality over time (p >0.05) [26]. We used SAS 9.3 (Cary, NC).

## Results

Of the 13,358 NHANES III adult (age ≥ 18 years) men and non-pregnant women who were examined and completed a periodontal disease assessment, we excluded participants due to missing data on serum creatinine (747), urine ACR (187), income (180), health insurance (561), hypertension (97), CVD (150), family history of CVD (200), HbA1c (58) or other covariates (423). Therefore, our final analytic sample included 10,755 individuals of which 1,335 met criteria for CKD. Compared with participants who were included in analyses, those who were excluded due to missing data were more likely to be older (mean age 49.3 vs. 41.5 years, p < 0.001); female (54.6 vs. 50.0 %, p < 0.001); and to have less than high school education (74.9 vs. 63.5 %, p < 0.001). There was no difference in racial/ethnic background distribution.

The overall prevalence of CKD was 12.4 % and the prevalence of periodontal disease was 7.6 %. Periodontal disease was significantly more common in participants who met criteria for CKD as compared to those who did not (9.2 vs. 4.8 %, p <0.001). The median follow-up time was 14 years.

Among individuals with and without CKD, those who had periodontal disease were more likely to be older, have annual family income < $20,000, and have less than high school education (Table 1). Participants with periodontal disease were also more likely to have less dental care visits, less annual physician visits; and were more likely to be smokers and have preexisting CVD and diabetes. Systolic BP was significantly higher among participants with periodontal disease compared with those with no periodontal disease, regardless of CKD status. Health insurance status was not associated with periodontal disease in participants with or without CKD. Among participants without CKD, the levels of HbA1c, total cholesterol, C-reactive protein, white blood cell count and plasma fibrinogen were statistically significantly higher in individuals with periodontal disease compared with those with no periodontal disease; however, given the magnitude of the absolute difference, the clinical significance is unclear. These differences in laboratory values were not statistically significant among participants with CKD (Table 1).

**Table 1:** Characteristics of NHANES III participants stratified by CKD AND periodontal disease status

	Overall N = 10755	CKD			No CKD		
		Periodontal Disease N = 193	No Periodontal Disease N = 1142	*P*	Periodontal Disease N = 625	No Periodontal Disease N = 8795	*P*
Characteristic	Weighted Mean (SE) or Weighted %						
Age, years	41.5 (0.5)	62.8 (1.6)	51.9 (1.2)	<0.001	53.5 (1.0)	39.6 (0.4)	<0.001
Female	50.0	42.2	62.1	<0.001	31.9	49.8	<0.001
Race							
Non-Hispanic White	75.7	69.5	73.5	0.4	67.2	76.4	0.004
Non-Hispanic Black	10.7	25.1	11.9	<0.001	16.9	10.2	<0.001
Mexican American	5.5	3.9	4.6	0.5	4.2	5.6	0.007
Other	8.2	1.5	10.0	0.001	11.7	7.9	0.1
Annual Income < $20,000	27.9	60.9	35.3	<0.001	39.9	26.3	<0.001
< High School Education	63.5	89.9	70.1	<0.001	77.8	61.8	<0.001
Health Insurance	86.5	88.8	89.7	0.8	85.9	86.2	0.9
Dental care use	71.0	49.5	70.10	<0.001	60.3	71.8	<0.001
Annual Physician visit	77.2	75.7	88.5	0.003	72.1	76.4	0.1
Never Smoked	48.4	25.0	46.5	<0.001	22.3	50.1	<0.001
Hypertension	20.4	44.3	42.2	0.7	28.5	17.7	<0.001
Diabetes	5.8	30.7	20.3	0.03	10.8	3.9	<0.001
Cardiovascular Disease	3.4	16.7	9.8	0.02	10.4	2.3	<0.001
Family history of CHD	9.2	13.2	9.7	0.4	12.2	8.9	0.2
Body Mass Index, Kg/m^2^	26.4 (0.1)	28.1 (0.7)	27.8 (0.4)	0.7	27.1 (0.2)	26.2 (0.1)	<0.001
Systolic BP	120.6 (0.4)	143.0 (1.9)	131.6 (1.0)	<0.001	128.8 (1.1)	119.0 (0.3)	<0.001
Hemoglobin A1c, %	5.3 (0.02)	6.4 (0.37)	5.9 (0.08)	0.07	5.7 (0.08)	5.2 (0.02)	<0.001
Total Cholesterol, mg/dL	200.3 (0.9)	220.3 (5.1)	213.3 (2.0)	0.3	214.9 (2.0)	198.1 (0.9)	<0.001
c-Reactive Protein, mg/dL	0.4 (0.01)	0.6 (0.05)	0.6 (0.04)	0.9	0.5 (0.04)	0.3 (0.01)	<0.001
White Blood Cell Count/μL	7.2 (0.04)	8.3 (0.34)	7.5 (0.13)	0.05	7.9 (0.16)	7.1 (0.04)	<0.001
Plasma Fibrinogen, mg/dL*	298.3 (2.7)	342.0 (8.0)	328.1 (6.5)	0.1	312.9 (6.1)	291.6 (2.7)	<0.001

*Plasma Fibrinogen was only measured in participants aged 40 years and older

Abbreviations: BP, blood pressure; CHD, coronary heart disease

Table 2 summarizes event rates and hazard ratios for all-cause and cardiovascular mortality, sequentially adjusted for the following variables: a) Model 1: age, gender, race/ethnicity, education, annual income; b) Model 2: Variables in Model 1 plus diabetes, hypertension, smoking, CVD, BMI, family history of premature CVD, HbA1c, total cholesterol and systolic BP. In fully adjusted models, as compared to participants with neither periodontal disease nor CKD, those with periodontal disease only or CKD only had increased risk of all-cause mortality (HR 1.39; 95 % CI, 1.06-1.81 and 1.55; 1.30-1.84, respectively). The presence of both periodontal disease and CKD was associated with HR, 2.07; 95 % CI, 1.65-2.59 for all-cause mortality and HR, 2.11; 95 % CI, 1.52-2.94 for cardiovascular mortality. Similar results were observed when we examined the joint effect of periodontal disease with low eGFR, and periodontal disease with albuminuria (Table 2). We did not find evidence of significant additivity or multiplicativity between periodontal disease and CKD on the outcomes of all-cause mortality (p value 0.8 and 0.9, respectively) or cardiovascular mortality (p value 0.9 for each).

**Table 2:** Cox proportional hazards models for all-cause and cardiovascular mortality evaluating the joint effect of periodontal disease and chronic kidney disease

	Events, N	Mortality Rate (1000 P-Y)	Model 1^a^, HR (95 % CI)	Model 2^b,^ HR (95 % CI)
All-cause Mortality				
(−) Periodontal Disease, (−) CKD	970	5.44	Reference	Reference
(+) Periodontal Disease, (−) CKD	228	21.33	1.67 (1.30,2.14)	1.39 (1.06,1.81)
(−) Periodontal Disease, (+) CKD	476	24.93	1.85 (1.57,2.18)	1.55 (1.30,1.84)
(+) Periodontal disease, (+) CKD	139	65.20	2.69 (2.14,3.40)	2.07 (1.65,2.59)
(−) Periodontal Disease, (−) Low eGFR	1182	5.98	Reference	Reference
(+) Periodontal Disease, (−) Low eGFR	293	23.80	1.65 (1.33,2.06)	1.39 (1.09,1.77)
(−) Periodontal Disease, (+) Low eGFR	264	56.78	1.58 (1.30,1.93)	1.39 (1.12,1.71)
(+) Periodontal disease, (+) Low eGFR	74	112.35	2.28 (1.72,3.02)	1.68 (1.20,2.36)
(−) Periodontal Disease, (−) Albuminuria	1140	6.13	Reference	Reference
(+) Periodontal Disease, (−) Albuminuria	268	23.29	1.63 (1.30,2.06)	1.40 (1.09,1.81)
(−) Periodontal Disease, (+) Albuminuria	306	19.44	2.12 (1.78,2.52)	1.65 (1.39,1.98)
(+) Periodontal disease, (+) Albuminuria	99	58.86	2.68 (2.06,3.49)	1.90 (1.47,2.46)
Cardiovascular Mortality				
(−) Periodontal Disease, (−) CKD	353	1.91	Reference	Reference
(+) Periodontal Disease, (−) CKD	84	7.60	1.59 (1.06,2.38)	1.33 (0.88,2.01)
(−) Periodontal Disease, (+) CKD	243	13.68	2.30 (1.89,2.80)	1.74 (1.41,2.15)
(+) Periodontal disease, (+) CKD	72	33.32	3.03 (2.18,4.21)	2.11 (1.52,2.94)
(−) Periodontal Disease, (−) Low eGFR	445	2.23	Reference	Reference
(+) Periodontal Disease, (−) Low eGFR	117	9.15	1.59 (1.19,2.12)	1.33 (0.99,1.79)
(−) Periodontal Disease, (+) Low eGFR	151	33.30	1.79 (1.33,2.41)	1.45 (1.05,1.99)
(+) Periodontal disease, (+) Low eGFR	39	55.04	2.21 (1.38,3.56)	1.45 (0.80,2.62)
(−) Periodontal Disease, (−) Albuminuria	450	2.35	Reference	Reference
(+) Periodontal Disease, (−) Albuminuria	106	8.59	1.51 (1.05,2.18)	1.38 (0.94,2.03)
(−) Periodontal Disease, (+) Albuminuria	146	10.07	2.56 (2.06,3.19)	1.78 (1.40,2.28)
(+) Periodontal disease, (+) Albuminuria	50	29.67	2.90 (1.91,4.43)	1.75 (1.12,2.73)

^a^Model1: Adjusted for age (continuous), gender, race/ethnicity (Non-Hispanic White, Non-Hispanic Black, Mexican-American or other), education (< vs. ≥ high school) and annual income (< vs. ≥ $20,000)

^b^Model 2: Adjusted for variables in Model 1 plus diabetes, hypertension, smoking (never vs. current/past), cardiovascular disease, BMI (continuous), family history of premature CVD, HbA1c, total cholesterol and systolic BP. In addition, analyses of periodontal disease and eGFR were adjusted for natural log of urine albumin-to-creatinine ratio; and analyses of periodontal disease and albuminuria were adjusted for eGFR

Abbreviations: CKD, chronic kidney disease (defined as either low estimated glomerular filtration rate [eGFR < 60 ml/min/1.73 m^2^] or the presence of albuminuria [urine albumin-to-creatinine ratio ≥ 30 mg/g])

In stratified analyses by CKD status, periodontal disease was associated with fully adjusted HR (95 % CI) 1.35 (1.04-1.76) for all-cause mortality and 1.36 (0.95-1.95) for cardiovascular mortality among individuals with CKD.

## Discussion

In a nationally representative sample of the U.S. population with 14 years of follow-up, our findings confirm the well-established association between periodontal disease and increased mortality in the general population, and provide new evidence of this association among individuals with CKD.

Over the past two decades, there has been accumulating evidence suggesting a link between periodontal disease and increased cardiovascular risk. In a recently published study of 1200 Veterans with median follow-up of 24 years, chronic periodontal disease was associated with an increased risk for incident coronary heart disease [9]. A number of other epidemiological studies have also reported an association between periodontal disease and cardiovascular events and all-cause mortality in the general population [6–8, 10, 11]. In contrast, this association was not found in the Physicians’ Health and Health Professionals Study [27, 28]. However, it has been speculated that the negative findings in these two studies may be due to the fact that periodontal disease was assessed by self-report rather than by a dental examination. Of note, most of the studies published to date evaluating the association between periodontal disease and outcomes have focused on general populations and therefore have not included a significant number of individuals with CKD.

Several cross-sectional studies have reported an association between periodontal disease and CKD. Analyses from the Atherosclerosis Risk in Communities Study and NHANES reported that periodontal disease was associated with a two-fold increased risk for prevalent CKD [13–15]. The cross-sectional design of these studies limits our ability to understand reasons for this association. It is possible that CKD leads to increased susceptibility to periodontal disease or that the chronic infectious and inflammatory milieu of periodontal disease may increase susceptibility to develop CKD. Alternatively, the relationship could be bidirectional as it has been proposed [16]. Two prior longitudinal studies have evaluated the association of periodontal disease with kidney function: a retrospective cohort analysis from 317 elderly men and women from Japan showed that during a 2-year follow-up, periodontal disease was associated with a greater than 2-fold risk of decreased kidney function; [29] and a study 529 individuals with type 2 diabetes followed by 22 years which reported higher incidence of end-stage renal disease in persons with moderate or severe periodontitis compared with those with none or mild periodontitis [30]. However, the association of periodontal disease and CKD with mortality has not been previously assessed. We hypothesized that there would be a significant joint effect of periodontal disease and CKD on all-cause and cardiovascular mortality, but we found this not to be the case. These findings might indicate that the mechanisms underlying the association between periodontal disease and mortality, and CKD and mortality, may be similar.

This represents the first prospective evaluation of the association of periodontal disease with mortality in persons with CKD. In stratified analyses restricted to participants with CKD, individuals with periodontal disease had 35 % increased risk for all-cause mortality as compared to individuals without periodontal disease, findings that are similar to what has been observed in studies of non-CKD populations. However, we found no significant association between periodontal disease and cardiovascular mortality which might have been due to lack of statistical power. The association between periodontal disease and mortality among individuals with CKD is of particular relevance because periodontal disease represents a potentially modifiable risk factor. Although there is some evidence that treatment of periodontal disease is associated with a reduction in markers of inflammation and endothelial function [31], the impact of intervention on clinical outcomes has not been studied.

## Conclusions

This study provides the first evidence that among individuals with CKD, periodontal disease increases the risk for mortality. Further studies are needed to assess whether treatment of periodontal disease can improve outcomes for patients with CKD. In the meantime, the increased prevalence of periodontal disease in CKD and its association with increased mortality risk suggest that general population recommendations for regular dental exams and oral hygiene should be emphasized among CKD patients.

### Strengths and limitations

The strengths of this study are long duration of follow up and a nationally representative sample. However, our study has several limitations. First, CKD was defined by single measurement of eGFR and albuminuria which could lead to misclassification. Second, periodontal disease status was only evaluated in one-half of the mouth which could lead to an underestimation of prevalence. Moreover, we based our periodontal disease definition on the criteria proposed by the CDC for use in population-based surveillance of periodontitis which might also underestimate the prevalence of periodontal disease [32]. Nonetheless, we still found a robust association with mortality. Third, periodontal disease was evaluated only once and therefore, we were unable to account for development of the disease during follow-up, exacerbations and treatment. However, interval periodontal disease treatment would have biased the results toward the null hypothesis.
